# Corrosion of care and disempowerment in acute psychiatry: As seen from the positions of therapists and suicidal patients

**DOI:** 10.1177/13634593241303617

**Published:** 2024-12-13

**Authors:** Julia Hagen, Birthe Loa Knizek, Heidi Hjelmeland

**Affiliations:** NTNU Samfunnsforskning AS (NTNU Social Research), Norway; Norwegian University of Science and Technology (NTNU), Norway; Norwegian University of Science and Technology (NTNU), Norway

**Keywords:** care, positioning theory, psychiatry, qualitative research, suicide

## Abstract

For people in suicidal crisis, referral to a psychiatric hospital is common. However, acute psychiatry is characterized by a lack of resources in terms of time and beds, making it challenging for therapists to provide person-centered care. In this qualitative study, we explored the experiences and positionings of therapists and suicidal patients in an acute psychiatric ward in Norway. We generated data through participatory observation and interviews with therapists and patients and analyzed the material using principles from Systematic Text Condensation supplemented with an analysis from a Positioning theory perspective. We developed two themes: *Therapists positioned as professionals with authority in a context with restricted action radius*, and *Patients in suicidal crisis positioned as medical subjects with limited influence*. In this resource-limited context, therapists managed their work and obligations by simplifying the patient’s suffering and suicidality and by emphasizing medical aspects. Ensuring an efficient patient flow was a high priority. The therapists’ authority and actions were closely connected to how patients were positioned and their experiences of the care. Positioning theory provides new perspectives for understanding the power imbalance in the positions of therapists and patients. The findings provide insights into acute inpatient psychiatry as a normative field where the choices and actions of both therapists and patients are restricted. In that sense, both patients and therapists can feel powerless. The findings point to significant limitations in the acute mental health care of people in suicidal crisis.

## Introduction

Annually, more than 600 persons take their lives in Norway. In the period 2010–2020, about 45% were in contact with specialized adult mental health- and substance abuse services during the last year before the suicide (Walby et al., 2023). About 10% took their life while admitted to inpatient mental health services; 32% of them during the first week, and 62% during the first month (Walby et al., 2023). It is unclear if or how the persons’ suicides may be related to their contacts with these services, but suicide prevention continues to be a challenge in mental health care. In psychiatric hospital wards, the understanding of suicidality and the approach to suicidal patients is strongly influenced by medical and psychological perspectives, particularly the former (Hagen et al., 2018). This means that professionals try to decrease suicidality and prevent suicide among patients largely by diagnosing and treating a presumed underlying mental disorder. Thus, suicidality is related to individual psychopathology (Marsh, 2010, 2016) and is largely medicalized (Pridmore, 2011). In the Western world, medical interventions have dominated suicide prevention, and some years ago, the Suicide Crisis Syndrome was proposed as a suicide-specific disorder (Galynker, 2017). An emphasis on individual traits and medical solutions implies that important aspects related to the suicidal person’s life and context are neglected (Hjelmeland and Knizek, 2017; Pridmore, 2011). The medical model has its limitations and might even be a misleading framework for understanding suicidality, and indeed, the assumed 90% association between mental disorder and suicidality is rebutted (Hjelmeland et al., 2012; Hjelmeland and Knizek, 2017). Some researchers and practitioners have even argued that the medical model might be an obstacle to a more person-centered and collaborative approach (Jobes, 2006/2016; Michel et al., 2016).

Our previous study of therapists’ (psychiatrists and psychologists) and mental health nurses’ experiences of caring for suicidal patients in light of ethics of care and ethics of justice indicated two approaches to these patients: “connection and care” and “duty and control” (Hagen et al., 2017a). The first is largely characterized by personal, relational, and emotional care, whereas the second is characterized by a more instrumental and formal approach to suicidal patients. The findings indicate that the two approaches and the ethical perspectives are intertwined. However, sometimes, ethics of justice may conflict with ethics of care when the professionals’ focus on medical aspects and procedures contributes to less flexibility in meeting with the patient and less focus on the person’s particular needs and wishes (Hagen et al., 2017a). This is a challenge in mental health practices increasingly shaped by standardization and guidelines, such as in Norway with for example the National patient pathway for mental disorders (Directorate of Health, 2023) and the National guidelines for the prevention of suicide in mental health care (Norwegian Directorate of Health and Social Affairs, 2008). The latter strongly emphasizes the assessment and management of suicide risk, and therapists spend much time assessing and documenting this (Hagen et al., 2017b). Emphasis on formal and instrumental procedures may limit the emotional care of the patient, which would be ethically questionable considering how important such care is for patients (Berg et al., 2017; Berglund et al., 2016; Hagen et al., 2018), and for preventing (new) suicide attempts.

This article is based on a field study in acute psychiatry, a project conducted in the period 2018–2020. In a previous article from this field study, we described how 11 patients’ (women) experiences of suicidality and hospitalization in a locked acute ward involved “liminality and weakened sense of personhood” (Hagen et al., 2020). The patients experienced being in a temporary painful state and place where they felt strong negative emotions, changed sense of self, and disconnection from themselves and others, in addition to being in a position with less autonomy. Further, the women’s experiences pointed to significant aspects of care that may strengthen suicidal patients’ personhood, such as the professionals recognizing the patients as equal and valuable human beings, showing respect, taking them seriously, listening to them, and understanding their needs (Hagen et al., 2020). Good connections between the suicidal patient and professionals in psychiatric wards, where the suicidal person and his/her experiences are acknowledged, are important for recovery (Berg et al., 2017; Hagen et al., 2018, 2020; Sellin et al., 2017). However, professionals are not always able to provide such care, or they do not have the opportunity to prioritize it (Berg et al., 2017; Hagen et al., 2017b, 2020). We need more knowledge about professionals’ positions and interactions with suicidal patients in a psychiatric hospital context and about structural conditions contributing to shaping professional-patient interactions and patients’ experiences of care.

Through Positioning theory, it is possible to study ways people construct and position themselves and others, both in action and speech. Positioning theory can contribute to increased understanding of interpersonal encounters and practices in different contexts by providing a framework for the comprehension of why individuals choose to act in a specific way out of many possible actions (Harré et al., 2009). The positioning of individuals and their actions is based on their specific beliefs regarding their action radius in the normative context of rights, duties, and obligations (Harré et al., 2009; Harré and Van Langenhove, 1999). The positions are flexible, but function as a compass in the determination of how to behave in certain situations. According to Van Langenhove (2021), “positioning refers to the processes of assigning, appropriating, or rejecting positions,” where every position determines possible actions. Further, the action radius of people is influenced by three factors: People’s capacity to do certain things, the restrictions imposed to do certain things, and the intentions people have to do certain things (Van Langenhove, 2021).

Drawing on Positioning theory, the aim of this study was to explore the experiences and positionings of therapists and suicidal patients in acute psychiatry. Our research questions were: How do therapists position themselves in relation to suicidal patients, and what do therapists emphasize to fulfill formal duties and obligations in an acute psychiatric context? How are patients in suicidal crisis positioned, and how do they experience meetings with therapists and care in this context?

We mainly use the term “psychiatry” rather than “mental health care” since this is the term used in the study context, and it reflects that psychiatry is, first and foremost, a medical specialty. However, we use mental health care/services when we refer to such services in general and when this term is according to cited articles. We use the term “suicidal patient/person” or “patient/person in suicidal crisis” interchangeably, although recognizing that the patients are unique individuals with different backgrounds and that they are not suicidal all the time. We consider suicidality as a complex, dynamic, and fluctuating process, influenced by the context and the person’s experiences and interactions with other people.

## Materials and methods

This study is based on ethnographic approaches (Angrosino, 2007; Fangen, 2010), and we generated and analyzed data based on qualitative methodology. We conducted the study from a social constructionist position, viewing knowledge as co-constructed, relational, contextual, and changeable (Gergen, 2015). Findings based on observations and individual interviews with the 11 patients have been published previously (Hagen et al., 2020) and we use some of the same data to explore this study’s research questions.

### Study context

We conducted the study in an acute psychiatric hospital department in Norway. The department consisted of several connected wards, providing acute care to people over 18 years of age admitted because of various mental health problems. We got permission to conduct the study in the two general acute wards, and not in the intensive care ward due to safety reasons (e.g. aggression among patients). The staff consisted of therapists (psychiatrists, specialist psychologists, psychologists, physicians, residents/physicians in specialization) and milieu personnel. Here, we focused on the therapists’ position and practice. In Norway, the therapists have the legal treatment responsibility of the patients, and the specialists (psychiatrists and specialist psychologists) have the most responsibility. Although the therapists have different professional backgrounds (medicine and psychology) the role of therapists is similar (except psychiatrists prescribe medication). However, when it comes to authority and power in this setting, there is a hierarchy within the therapist group. The chief psychiatrist has the most power, followed by specialists (psychiatrists and specialist psychologists), and then non-specialists (psychologists, physicians, and residents/ physicians in specialization). The patient had one or two primary therapists during their stay, and at least one of them was a specialist.

### Participants and recruitment

The sample consists of 20 participants: 9 therapists (referred to as T1–T9) and 11 patients admitted because of suicidality (referred to as P1–P11). The patients are numbered according to the order in which they were recruited. The nine therapists (six men, and three women) participated in the observational part of the study: Four psychiatrists, one specialist psychologist, one psychologist, one physician, and two residents/physicians in specialization. Thus, the therapists were five specialists (T2, T3, T4, T8, T9) and four non-specialists (T1, T5, T6, T7). Four of the nine therapists (T1, T2, T3, T4) participated in both the observational part of the study and one focus group interview. These were three men (one psychiatrist, one specialist psychologist, one psychologist) and one woman (psychiatrist), aged 36–55 years, with working experience in psychiatry ranging from 5 to 19 years.

The 11 patients (women, aged 21–41 years, median age 33 years) participated in both the observational part of the study and one individual interview. The participants were voluntarily admitted to the acute psychiatric ward after self-harm/suicide attempt (*N* = 3) or suicidal thoughts (*N* = 8). Five participants were admitted to a psychiatric hospital for the first time, two had been admitted 1–3 times before, whereas four had been hospitalized more than 10 times over several years. They were hospitalized for 1–3 days (*N* = 2), approx. One week (*N* = 4), or several weeks (*N* = 5). Two participants were transferred to a district psychiatric center but returned to the acute ward shortly after. The three participants who had the longest stay in the ward were still hospitalized when JH completed the data generation.

The sample of participants is purposive, and we recruited therapists and patients based on their characteristics and experiences according to the study’s aim (Patton, 1990). The strategy for purposefully selecting participants was influenced by both criterion sampling (characteristics) and convenience sampling (accessibility) (Patton, 1990). JH recruited therapists by approaching therapists in the ward who had responsibility for suicidal patients and invited them to participate. Of the nine included therapists, the five most experienced ones were invited to participate in a focus group interview. Four of them agreed to participate. JH recruited patients with the assistance of therapists, who decided whether it was appropriate to invite the individual patient to participate at that moment. A few patients were not invited to participate because the therapists considered them too vulnerable, and participation was considered an additional burden for them.

### Data generation

The first author JH has a background as a mental health nurse and researcher in the field and spent approximately 160 hours (during a period of 3 months) in the acute ward during day shifts (7 a.m. to 3 p.m.). In this period, JH generated data through participatory observation and interviews. Participant observation involved taking part in some of the activities in the ward and included observation of one or two of the patients’ meetings with their therapists, observations of how therapists acted and what they said, and conversations with individual therapists and patients. These conversations were less structured than the interviews and were not audiotaped. Most conversations with individual therapists occurred after their meetings with patients, in one of the offices in the ward. JH had more conversations with the patients than the therapists, having one to four conversations with nine of the women. Most of these conversations were in the patients’ room, where they felt most comfortable talking about their situation. Some conversations occurred in one of the living rooms in the ward. JH wrote field notes from the observations and conversations (usually in one of the offices in the ward), and the most extensive notes and reflections were written at the end of each shift. In addition to observations and conversations, JH conducted one focus group interview with 4 therapists and individual interviews with 11 patients.

The focus group interview with the therapists was conducted at the end of the data generation period after the interviews with patients were completed. It took place in a meeting room outside the ward. JH used an interview guide as a tool to help structure the interview, with the following main questions: What do you think is important to be able to provide good treatment and care for suicidal patients? What is your most important task? What do you think is important to prevent suicide/suicide attempts among patients? Are there any conditions that may contribute to challenging your work and your treatment and care for suicidal patients? Do you have any suggestions for changes that can help improve treatment and care for these patients? The strength of a focus group is the dialog between the participants (Malterud, 2012), but it was a bit difficult to facilitate the dialog between these participants. The interview lasted for 70 minutes and was audiotaped and transcribed.

Individual interviews with the patients were conducted on the same day they were recruited, or the following day, in different meeting rooms in the ward. JH used an interview guide to help structure the interview, with the following main questions: Can you please tell me about your situation and the reason you came to the acute ward? How do you experience the meetings with the therapists and nurses? Can you please give examples where you did/did not feel well taken care of by the therapist/nurse? What is most important for you to get better, including less suicidal thoughts? In keeping with Kvale and Brinkmann (2009) JH probed for further elaboration (e.g. Can you please tell me more about. . .?) to obtain specific and rich information about the patients’ experiences. The interviews lasted from 30 to 85 minutes, and all except one were audiotaped and transcribed. One interview was not audiotaped because the patient did not want to, and hence, JH wrote notes during this interview. These notes were added to the rest of the data material consisting of field notes from the observations and conversations and the transcripts from the interviews.

### Analysis

We analyzed the data using principles from Systematic Text Condensation (STC) (Malterud, 2017), supplemented with an analysis from a Positioning theory perspective (Harré and Van Langenhove, 1999; Van Langenhove, 2021). STC is a systematic method for identifying patterns and developing themes across qualitative data. The approach is suitable for exploring subjective experiences and positions in different contexts and from different perspectives, and it is encouraged to use theory to sharpen the focus of interpretation (Malterud, 2016, 2017). The method of analysis implies that there can be several alternative interpretations at the same time (Malterud, 2017), thus, supporting a social constructivist position where knowledge is considered contextual and changeable - there is no “one truth” (Gergen, 2015).

The first author conducted all steps of the analysis. The second and third author read the transcripts from the interviews and the relevant parts of the field notes and participated in the analysis to provide more depth and nuances in the interpretations. The analysis was conducted in the following (simplified) four steps: (1) JH read the field notes and interview transcripts several times to get an overall impression of the material and identify preliminary themes (studying what is focused upon in acute psychiatric care, organization of care, working conditions, experiences of care). (2) JH extracted meaning units from the material and sorted them into codes (e.g. establishing relationships, treating underlying disorders, suicide risk assessments, documentation requirements, lack of time, and more). (3) JH sorted the codes into preliminary code groups, e.g. “psychiatrization” of suicidality, acute psychiatric care under pressure. During this process, we had several meetings to discuss the material and the preliminary findings, and we found that the therapists’ and patients’ experiences and interactions could be understood in terms of positionings and positions. (4) We then used the Positioning theory (Harré and Van Langenhove, 1999; Van Langenhove, 2021) to further develop our analysis: studying the interview transcripts, field notes, preliminary themes, and code groups developed in the initial phase, focusing on how the therapists were positioned or positioned themselves in relation to suicidal patients, and how the patients were positioned by therapists and how this could influence on the patients’ experiences of care. We had several meetings, discussed our findings, and moved back and forth between the data, our preliminary findings, and the theory until we agreed upon the final two themes.

### Ethical considerations

This study was conducted in keeping with guidelines set by The National Committee for Research Ethics in the Social Sciences and the Humanities (2022). The Regional Committee for Medical and Health Research Ethics approved the study (2018/932). The participants signed one or two informed consent forms, one for allowing the researcher to conduct participatory observation in the ward (all participants) and one for participating in a focus group interview (four therapists) and individual interview (11 patients). JH informed the participants that they could withdraw from the study at any time before publication without giving any reason. We treat the data confidentially and present information about the therapists and the patients in a way that they are not identifiable to others. In the findings, therapists and patients are identified by numbers (T1, P4, and so on), and therapists are referred to as “he.” The findings are substantiated by extracts from JH’s observations and conversations with the participants and quotes from the participants.

## Findings

We developed two themes: *Therapists positioned as professionals with authority in a context with restricted action radius*, and *Patients in suicidal crisis positioned as medical subjects with limited influence.* The first theme is closely connected to the second theme, since the positioning of the therapists and their practice influenced on how suicidal patients were positioned and how they experienced care.

### Therapists positioned as professionals with authority in a context with restricted action radius

Our findings indicate that the therapists positioned themselves or were positioned as professionals with authority, who displayed competence, confidence, power, and efficiency in interactions with patients. Several patients talked about therapists as professionals with authority and power, and one patient (P4) put it this way: “. .they [therapists] are the ones who have all the power, they are the ones who decide what to do.” She had been hospitalized several times and sometimes felt she had little influence on the course of treatment. During observations of therapists’ practice and interactions with patients, JH noticed that even therapists with limited experience in psychiatry and this ward appeared to be positioned as professionals with authority. For example, after a 10-minute meeting with a patient who was going to be discharged, the therapist (T6) confidently explained the reason for the discharge: “I can’t see any objective signs of depression, anxiety or mental illness. . and I had expected that the patient had taken more responsibility.” Thus, it seemed as if objective signs were emphasized over the patient’s subjective experiences. The therapist’s narrow focus appeared related to this treatment context and indicated that therapists had a restricted action radius, suggesting that their authority had its limits.

The researcher observed that the therapists’ practice was influenced by a high workload and scarcity of beds and time, and the work conditions and obligations seemed to contribute to what sometimes appeared as superficial approaches to patients’ often complex challenges, which the abovementioned therapist’s (T6) emphasis on objective signs illustrated. To manage the workload and ensure efficient patient flow, it seemed like the therapists simplified and emphasized certain aspects of the patient and the situation so the patient could be discharged or transferred to another unit as quickly as possible. When JH posed the following question to the four therapists in the focus group: “What is your most important task when the suicidal patient is admitted to the acute ward?,” one of them (T1) answered:

Primarily, point 1: to find, in a way, the most appropriate treatment outside the house [ward], i.e., where the patient should go next. What will, in a way, provide the best care? I also think, point 2: adequate treatment, in a way, according to the patient’s diagnosis. I don’t necessarily think that one always should treat the suicidality per se, or in itself, but that one can, in a way, treat, – in quotation marks – the underlying disorder, whether that is depression or emotional instability or whatever it is. Mmm. Briefly summarized.

The therapist seemed confident, competent, and efficient—briefly and clearly summing up what he thought was a priority in approaching the suicidal patient, and hence, he seemed to position himself according to perceptions of the duties in this context. Seeking other treatment options and planning the discharge started at the time of admission, and the statement reflects the importance of this, as it was mentioned first, and before the therapist talked about the treatment of the individual. Further, identifying and treating an assumed underlying mental disorder was prioritized, also related to reducing suicidality. Although, when the therapist said, “quotation marks” before “underlying disorder,” it indicates some uncertainty about the understanding of an “underlying disorder,” whether there always is such an underlying *illness* present, or perhaps an awareness of disorders’/diagnoses’ limitations when it comes to understanding a person’s suicidality. Still, identifying and treating an assumed underlying disorder may be a way of restricting the focus to what was considered manageable entities (diagnosis), and it appeared as a practical and pragmatic approach according to obligations and expectations.

Several therapists said they emphasized relational aspects of care, such as building alliances with the patients and having time to listen to understand them, as illustrated by this therapist (T2): “In concrete terms, I think, I need to have time and some space to talk with those patients, to, in a way, be able to both see them and understand them.” Often though, time pressure and duties according to policies seemed to push them into a narrower medical perspective. JH observed that there was a focus on medical aspects in several therapist-patient meetings, where the therapist dominated the talk and focused mostly on the patient’s symptoms and the medical treatment (medication and/or Electroconvulsive therapy - ECT). After one such meeting (T5 and P7), JH asked the therapist whether he had considered other and non-medical aspects and interventions, for example by exploring the patient’s home situation. The therapist replied: “She is too ill.” He further explained that the patient had to get better from her depression by receiving more ECT and medication before being ready to go into such aspects. The therapist’s approach and interaction with the patient appeared superficial, yet it also appeared to be an effective way of managing obligations according to policies in this context. Thus, it seemed as if a positioning according to perceptions of duties and obligations was functional. The therapists appeared to find ways of managing their work efficiently, but that did not mean it was always easy. For example, making assessments related to discharge appeared demanding, especially when there was a suicide risk. One therapist (T4) shared this experience in the focus group:

What I think can be complicated in the acute ward is that you constantly have to push things, to have the fastest possible discharge. . . (. . .) . . .so you have to try to push it as much as possible. And that, it means that every once in a while, when you push it so much, then things happen. So, it’s a. . .it’s a thing I often think about, how far can you push it, with regard to. . . (. . .) it might just as well be discharges, because you discharge people who say they are going to take their own lives, just like you. . . yes, you don’t admit people who say they are going to take their own life. You have to make an assessment there and, of course, if it is chronic suicidality, so. . .yes, then you let it pass, as long as it is not [chronic suicidality].

The statement illustrates the pressure to work fast—*to push things*—to ensure efficient patient flow. Further, it indicates that when therapists push it too much, for example by discharging the patient earlier than planned, or before the person is ready to leave the hospital, suicidality may increase, and the person may attempt suicide. To the very least, this therapist seemed to worry about whether this could happen. The therapists’ duty to ensure the fastest possible discharge seemed to position them as gatekeepers of this service, thus distributing rights to inpatient acute care among people. On a few occasions, JH observed that the therapists’ authority was diminished by the chief psychiatrist, who ordered therapists to discharge patients even if they thought it was too early. Thus, the chief psychiatrist was positioned with more authority and power than the therapists, and as a professional who could restrict the therapists’ action radius further. In the focus group, two therapists (T1 and T4) stated that, sometimes, patients were more or less thrown out of the ward. One therapist (T4) elaborated:

Of course, there is pressure, and that. . (. . .) what is a good patient pathway then, concerning emotionally unstable [patients], is to say to your colleague: “Yes, I will throw her out.” But you don’t do that. You have a good discharge conversation. Because then everyone is happy. (. . .) . . ’throw out’, that sounds a bit harsh, and that’s what you should do, but then you don’t do it. You make a good discharge conversation. And then everyone is happy. Because it’s a bit like that. . . it’s a bit like. . . you hear that term, now you [researcher] are not at those meetings [therapists’ meetings], but that term is used a lot.

The statement illustrates some of the challenges of being a therapist within a rather rough environment. The therapist’s reflection on the term *throw out* shows that he was a bit uncomfortable with it, although he acted according to his duty. Even though the therapists had limited choices in this situation, these two therapists emphasized conducting the discharge conversations in such a way that the patient could feel understood. Thus, they made efforts to remedy what can appear as inflexible conditions for both therapists and patients.

### Patients in suicidal crisis positioned as medical subjects with limited influence

Our findings indicate that in contrast to the positioning of the therapists, which reflected their duties and obligations in the care of suicidal patients, the positioning of the patients was related to the patients’ rights as service recipients and could serve as a justification for their right to be admitted to and stay in the acute ward. As already touched upon in the first theme, the suicidal patients seemed to be positioned as individuals subjected to medical authority, where the therapists’ emphasis on medical aspects involved positioning the patients along the same lines of reasoning – as medical subjects with limited influence.

First, we return to the therapist-patient meeting described above (T5 and P7), to provide more understanding of this interaction and the therapist’s focus on the patient’s symptoms/medication/ECT and the patient’s response. During the meeting, JH observed that the patient listened attentively and appeared compliant with the therapist’s agenda. Thus, the patient seemed to accept being positioned as a medical subject. Perhaps such a subordinate positioning could be of some relief for the patient, as it implied allowing herself to be *ill* and leave the responsibility to the therapist who after all had the duty to provide what was considered appropriate treatment and care. In the interview, the patient also said that she trusted the therapists she met when she was admitted to the ward: “ . .then I realized that these [therapists] know. . .know how I feel and what kind of help I need. So, I trusted them a lot then, and I still do.” She was admitted to an acute psychiatric ward for the first time and was positive about the treatment she received.

In JH’s reflection with the therapist (T5) after the meeting, when the therapist said that the patient was too ill and had to get better from her depression before being ready to talk about her home situation and other circumstances, the therapist positioned the patient incapable of dealing with negative experiences or deemed that it would be too stressful for her. Being positioned as the professional with authority, the therapist made that choice on behalf of the patient; a choice over which the patient seemed to have little influence. During the interview and several conversations, the patient (P7) had shared experiences from a stressful life situation with the researcher. In the last conversation with her, before she was transferred to another unit (after about 3 weeks), she stated that she was anxious about how it would be to come home again. She confirmed that her home situation had not been a topic in the acute ward, and she expected it would be addressed in the unit she was transferred to. She wanted changes in her life, and she wanted professionals to give her tools for how to cope with life. It seemed like the patient had been ready to talk about and deal with the circumstances that contributed to the suffering and suicidality. However, being a patient in suicidal crisis and admitted to an acute psychiatric ward for the first time, she appeared vulnerable, and she seemed to easily accept the subordinate positioning in relation to the therapist and to be at the mercy of the therapist’s approach. Even if that meant leaving significant needs unmet.

There were other examples of therapist-patient interactions illustrating more resistance from the patients, indicating that they did not accept, and even tried to challenge or reject, being positioned as medical subjects with limited influence. For example, after one therapist-patient meeting (T6 and P9), the patient said to JH: “He [therapist] does not listen to me.” She looked down, cried a bit, and seemed dejected. During the meeting, JH noticed that the therapist followed his own agenda. The therapist seemed to perform his work according to routines and policies, but he failed to respond to the patient’s needs to feel heard and understood. JH observed that the patient tried several times to assert her own needs, while the therapist returned to his agenda. The therapist appeared to control the content and pace of the conversation, and the patient seemed frustrated. JH further noticed that the therapist’s approach seemed a bit contradictory; he put pressure on the patient to accept his plan, but at the same time stated that she could decide for herself. Indirectly, it seemed like the patient had to comply with the therapist’s plan, thus, it was a negotiation about the course of treatment in which the patient seemed to lose. The patient being in the subordinate position, had to submit to the therapist with authority.

During one conversation, the patient (P9) said she felt fragile, but in the acute ward, she learned that things must happen quickly. She felt pressured and challenged and would like to do it more on her own terms. The patient felt she had little influence on her course of treatment, and she felt that treatment and care were more according to the needs of the system and professionals than according to hers. Several patients shared examples where they did not feel seen and heard, and sometimes they felt powerless with limited autonomy. However, the patients were reluctant to or did not want to express their dissatisfaction to the professionals in fear of conflict or other negative consequences. One patient (P10), who had negative experiences from previous stays in the acute ward pointed to the difficulty of standing up for herself:

. . because when you are here, you are locked in, you don’t decide by yourself whether you go out, and you don’t decide by yourself whether to be discharged, and they can define what they want within a frame where it is not visible to many others. And I have had experiences before that things went wrong then (. . .) if things go wrong, the communication gets so bad then, and then you are in it and have nothing to say or nothing to do.

The patients’ experiences illustrate their vulnerability of being a patient in this setting, and it indicates some costs of receiving care in a highly resource-limited treatment context, and of being in a position subjected to medical authority.

Several patients seemed aware of the limitations of resources in the acute psychiatric ward, such as lack of time. Some of them experienced treatment and care to be rushed at a pace that was too quick for them, including being pushed out of the ward or transferred to another unit before they felt ready. As one patient (P9) put it: “He [therapist] takes shortcuts.” Although the patients were dissatisfied with this, they appeared to realize the therapists’ pressure to work quickly—to *push things*, as one of the abovementioned therapists said. It was, however, uncertain whether the patients realized how much the therapists’ emphasis on categorization of the severity of symptoms and diagnosis influenced treatment and care and length of their stay. As suggested in the first theme, in the therapists’ distribution of rights to inpatient acute care, patients experienced as “emotionally unstable” were deemed as not “ill” enough to be hospitalized for more than a few days, or not at all. Regardless of diagnosis, several patients expressed the need for more time and a different kind of care than what they received, as illustrated by this patient (P4): “I need time. And I understand very well that it is. .that is the way it should be, that there are reasons why it is like that, but it is not, it does not work for everyone.” She further stated that such acute care was like “firefighting,” which was of little help and could lead to readmissions. Being met and recognized as an equal fellow human being where professionals tried to understand their pain and not only make judgments based on the diagnosis was reported as important for the patients: “Just being seen as a human being, really, and not being seen as a diagnosis, and not that they just see the act, because. . .just getting a little understanding and a little empathy, it can do so much.” (P4). It seemed as if she did not expect that much. Our findings indicate though that the room for individualized care is restricted in the acute psychiatric ward, and the patients’ desire for equality in the relationship with the therapists seemed difficult to achieve, even unrealistic, given the inflexible conditions contributing to shaping therapists’ practice and their interactions with patients.

## Discussion

This study provides insights into the experiences and positionings of therapists and suicidal patients in an acute psychiatric ward. Positioning theory (Harré et al., 2009; Harré and Van Langenhove, 1999; Van Langenhove, 2021) contributes to an increased understanding of therapists’ action radius in this specific context and the theory is useful for shedding light on the positionings of therapists and patients in relation to each other. Through the lens of Positioning theory, our findings particularly demonstrate a power imbalance in the positions of therapists and patients and the different ways in which this was expressed. Through the therapists’ authority and emphasis on diagnosis, symptoms, and medical treatment, the patient was positioned as a medical subject with limited influence on how their suffering and suicidality were understood and approached. The therapists could largely determine the patients’ course of treatment, and being in the subordinate position, the patients had to comply with their decisions. Although some patients tried to resist the therapist’s understanding and decisions, they were reluctant to express their disagreement or dissatisfaction in fear of negative consequences. By using Positioning theory, we have been able to illuminate mechanisms of power in therapist-patient relationships and illustrate how being in the subordinate patient position could contribute to patients feeling powerless.

Our study demonstrates how the medical model, and “the diagnosis-EBP [evidence-based practice] symptom-reduction system” (van Os et al., 2019: 93), permeated the therapists’ understanding and approach to (suicidal) people admitted to the acute psychiatric ward. The therapists’ obligations according to policies and the scarcity of time and other resources appeared to strengthen this emphasis. Thus, the therapists’ work conditions seemed to restrict their action radius and contribute to what sometimes appeared as superficial approaches to patients’ often complex challenges. The therapists’ actions and the positionings of therapists and suicidal patients can be understood in view of the theory of Street-level bureaucracy (Lipsky, 2010). This theory deals with how front-line professionals in public services experience challenges due to huge work requirements and a lack of resources necessary to provide individualized service. Inadequate resources make it necessary for professionals to simplify the cases and develop routines to manage the job according to policies, which may contrast with the individual needs of the service recipients (Lipsky, 2010).

Our findings demonstrate what Lipsky (2010) describes as the built-in contradiction involved in street-level bureaucrats’ work; they are expected to exercise discretion in response to individuals’ needs, while in practice, they must deal with people in terms of procedures, routines, and labels and other mechanisms to manage the work tasks according to obligations. As street-level bureaucrats, the therapists managed their work according to policies, obligations, and routines—in which ensuring an efficient patient flow was a high priority—but they sometimes failed to meet the patients’ need to be heard and understood.

Although Lipsky (2010) argues that professionals have discretion at their disposal, our findings indicate that since the therapists’ action radius was restricted in this setting, the space for discretion, and thus their possibility to provide person-centered care, was limited. In that sense, both the theory of Street-level bureaucracy and the Positioning theory are useful for understanding that not only patients, but also therapists can feel powerless within a resource-scarce health system that strongly regulates their practice and interactions with patients.

In a study exploring practice in a community mental health service through the lens of Lipsky’s theory (Bell and Hill, 2023), the authors state that although practitioners have a significant amount of discretion, mental health practice is shaped by neoliberalism and market-based reforms, which have led to increased focus on risk management, accountability and managing scarce resources. These conditions have narrowed the discretionary spaces, which the findings in our study also illustrate. The challenges and constraints due to neoliberalism and the medical model may be an obstacle to person-centered, relational, and compassionate care of people in suicidal crisis (Fitzpatrick and River, 2018; Jobes, 2006/2016; Michel et al., 2016). However, relational and emotional care according to their needs is what suicidal patients seek (Berg et al., 2017; Berglund et al., 2016; Hagen et al., 2018, 2020).

Although some patients/former patients have also shared positive experiences of treatment and care in psychiatric wards (Hagen et al., 2018, 2020), this study indicates that acute psychiatric care, as currently organized, may not necessarily be the best option for many of those experiencing a suicidal crisis. In keeping with Fitzpatrick and River (2018), it is a treatment option with major limitations that may not meet the needs and expectations of people in suicidal crisis. Hence, we need to consider developing other types of acute mental health care and suicide crisis care. For example, the Maytree Respite Centre in London offered up to 4 nights of non-medical support to people in suicidal crisis (Briggs et al., 2007, 2012). Briggs et al. (2012) found that most guests benefited from their stay at Maytree, some even experienced it as “transformational” (p. 9), contributing to reduced suicidality, hope, and changes in their lives. Inspired by Maytree, a similar low-threshold suicide crisis care center has opened in Oslo, Norway; “Livslosen.” We need more such services and other low-threshold support systems in the communities, where the primary aim is to contribute to the person feeling heard, recognized, and understood. Further, persons with lived experience have an important role in the design and delivery of alternative service models that meet the needs of persons in suicidal crisis (Fitzpatrick and River, 2018).

Other researchers and practitioners have highlighted the importance of collaborative and narrative approaches to people in suicidal crisis (Jobes, 2006/2016; Michel and Valach, 2011; White and Morris, 2019). Such approaches require the practitioner to establish a trusting relationship with the patient and to listen to the patient’s suicidal story (Jobes, 2006/2016). A narrative approach requires being open to the patient’s story and joining the person in his/her experience of suffering, an approach that may contribute to significant change: *When the story is told and retold to a sensitive listener the endpoint may change from death orientation to life orientation* (Michel and Valach, 2011: 71). Within a neoliberal and medical framework, certain stories and identities become available (e.g. the suicidal person is mentally ill and needs hospital treatment), whereas from a narrative viewpoint, several stories and identities are available (White and Morris, 2019). Through narrative questioning and mutual exploration of the person’s problems as well as abilities and hopes, the practitioner creates openings to life-promoting stories and identities (White and Morris, 2019). Adopting such an approach in acute mental health care has the potential to empower individuals, and their families, as they strive to find their way forward.
